# Hsp74, a Potential Bladder Cancer Marker, Has Direct Interaction with Keratin 1

**DOI:** 10.1155/2014/492849

**Published:** 2014-06-23

**Authors:** Ling Chen, YaRong Wang, Le Zhao, Wei Chen, Chunhui Dong, Xinhan Zhao, Xu Li

**Affiliations:** ^1^Department of Oncology, The First Affiliated Hospital of Xi'an Jiaotong University, Xi'an 710061, China; ^2^Department of Radiology, Tangdu Hospital, The Fourth Military Medical University, Xi'an 710038, China; ^3^Translational Medicine Center, The First Affiliated Hospital of Xi'an Jiaotong University, Xi'an 710061, China

## Abstract

Early diagnosis and prognosis monitoring are very important for the survival of patients with bladder cancer. To identify candidate biomarkers of bladder cancer, we used a combination of techniques including 2-DE, co-IP, western blot, LC-MS/MS, and immunohistochemistry. Hsp74 was identified with high expression in bladder cancer. The cellular location of expression products of gene Hsp74 showed that they were distributed into cytoplasm and keratin 1 was found to be associated with Hsp74. The results provide a new idea to understand the molecular basis of bladder cancer progression and pinpoint new potential molecular target for early diagnosis and therapeutic monitoring of bladder cancer.

## 1. Introduction

Bladder cancer is the 7th most common cancer in men and the 17th most common cancer in women in the world [1]. Occupational risks, environmental risks, dietary habits, and cigarette smoking are lifestyle factors influencing the development of bladder cancer. It is a common malignancy requiring a high degree of surveillance because of the frequent recurrences and the poor clinical outcome of invasive disease. Hematuria is often the onset symptom of bladder cancer; therefore, cytologic analysis of urine becomes the initial evaluation method, followed by cystoscopy with or without biopsy [2]. However, cytologic analysis has a limited value because it is operator-dependent and has low sensitivity. Cystoscopy still has some limitations [3, 4]; for example, it is invasive, time-consuming, and expensive, requires sedation or anesthesia, and sometimes leads to iatrogenic injury. Also, evaluation of lesions located in the base or neck of the bladder or in the diverticulum is difficult because of the limited perspective of the cystoscope [5, 6]. To date, serum biomarkers for bladder cancer have some practical value, but they lack optimal sensitivity and specificity in diagnosis and disease categorization. Recently, many radiologic imaging techniques have been used to detect and evaluate bladder tumors, but none is reliable in detection of bladder cancer. Therefore, it is important to establish early detection methods with high sensitivity and specificity for bladder cancer.

In this study, Hsp74 as a potential tumor antigen was identified by 2-DE and western blot using bladder cancer cell line BLZ211. Moreover, keratin 1 as an associated protein with Hsp74 was found by coimmunoprecipitation. These two molecules, in conjunction, might play a certain role in the progression of bladder cancer and might be seen as potential therapeutic target, which is more inspiring, though further investigations are needed. In addition, these data represent the first report, to our knowledge, of a functional link between Hsp74 and keratin 1 in bladder cancer cells.

## 2. Materials and Methods 

### 2.1. Cell Line and Preparation of Monoclonal Antibody (McAb)

Established bladder cancer cell line BLZ-211 was described previously [7–9]. Cells were maintained in RPMI-1640 supplemented with 10% fetal calf serum, 100 U/mL penicillin, and 100 *μ*g/mL streptomycin, at 37°C in a humidified atmosphere of 5% CO_2_. BLZ211 cells were harvested from monolayer cultures by trypsinization and the cell pellet was washed with sterile PBS and suspended in the same medium. Approximately 6 × 10^6^ cells were injected i.p. into 6-week-old female BALB/c mice. The animals were given 3 i.p. booster injections a week apart and final injection 3 days prior to fusion experiment. Spleen cells from these mice were fused with Sp2/0 myeloma cells (spleen cells versus myeloma cell = 5 : 1). 2-3 weeks after fusion, culture supernatants were analyzed using ELISA method for antibody production. Positive clones were selected and subcloned twice by semisolid cloning.

### 2.2. Two-Dimensional Electrophoresis

1 × 10^7^ BLZ211 cells were suspended in 200 *μ*L of lysis buffer (7 M urea, 2 M thiourea, 4% CHAPS, 0.5% Triton X-100, 0.5% IPG buffer, 2 mM TBP, and 50 mM DTT) supplemented with protease inhibitor cocktail and vortexed at 4°C for 1 h, and then the cell lysate was clarified by centrifugation at 40,000 ×g for 1 h. The supernatant was collected and protein concentration was determined by Bradford Assay using BSA as a standard. Two gels were prepared at the same time. An IPGPhor apparatus (Amersham-Pharmacia Biotech, Uppsala Sweden) was used for IEF with 13 cm pH 3–10 no-linear or pH 4–7 immobilized pH gradient (IPG) strips (GE Healthcare Bio-Science AB) at 20°C.The strips were rehydrated overnight with 250 *μ*L of rehydration buffer (7 M urea, 2 M thiourea, 4% CHAPS, 0.5% Triton X-100, 0.5% IPG buffer, 2 mM TBP, and 30 mM DTT) containing 50 *μ*g proteins. Isoelectric focusing protocol was followed as (1) 60 V, 12 h, Step and Hold mode; (2) 200 V, 2 h; Step and Hold mode; (3) 500 V, 1 h, Gradient mode; (4) 1000 V, 1 h, Gradient mode; (5) 8000 V, 1 h, Gradient mode; (6) 8000 V, Step and Hold mode until 30 kVhT was reached. Strips were then equilibrated in 10 mL equilibration buffer (6 M urea, 30% w/v glycerin, 4% w/v SDS, 50 mM Tris-HCl pH 8.8) with 1% w/v DTT for 15 min at room temperature. Strips were removed and incubated in equilibration buffer with 2.5% w/v iodoacetamide for another 15 min. After equilibration, the strips were embedded onto an 11%, 1 mm SDS/PAGE gel and were fixed in place with a 0.5% w/v agarose overlay. Gels were run in a SE600 (Amersham-Pharmacia Biotech, Uppsala Sweden) at 10 mA/gel for 20 min then 20 mA/gel until the bromophenol blue dye reached the bottom of the gel.

### 2.3. Western Blot

One of 2-DE gels with separated proteins were immunoblotted onto nitrocellulose membrane (0.45 *μ*m pore size; Bio-Rad) and blocked overnight at 4°C in SuperBlock Blocking Buffer in Tris-bufferedsaline (Pierce) to block the nonspecific bindingsite. As first antibodies, the MAb diluted 1 : 10 in blocking buffer and the membranes were incubated for 1 h at room temperature. After one hour incubation at room temperaturewith the second antibodies, the immunoproducts were visualizedwith ECL western blotting detection reagents (AmershamBiosciences) and exposure to X-ray films. Each wash was performedwith Tris-buffered saline-Tween (10 mmol/L Tris-HCl (pH 7.6), 100 mmol/L NaCl, and 1 mL/L Tween 20).

### 2.4. Silver Staining and Image Analysis

One of 2-DE gels was fixed in 30% alcohol containing 1% acetic acid for 2 h and was sensitized in 30% alcohol containing 0.2% Na_2_S_2_O_3_ and 6.8% sodium acetate for 30 min. The grids were removed with plastic forceps and washed by 3 immersions of 5 min each in double-distilled water. The gel was stained in 0.25% silver nitrate for 30 min. The grids were washed by 3 immersions of 1 min each in double-distilled water. 2-DE gel was developed in 0.74% formaldehyde containing 2.5% Na_2_CO_3_ for 4–8 min. At clear spot times, add 5% acetic acid for 10 min. The grids were washed by 3 immersions of 5 min each in double-distilled water. Gels were scanned with a ScanMaker 8700 scanister (MICROTEK, China). The images were saved in TIFF format and then exported to the ImageMaster 2D platinum Software 5.0 (Amersham-Pharmacia, Sweden). Spot detection parameters were best adjusted using, first, the Smooth parameter which was set to a value of 1.5 allowing the detection of all real spots and split as many as possible overlapping spots. Then, the minimum area was set to eliminate spots that have an area smaller than 15 pixels. Finally, the saliency parameter was experimentally adjusted to 1 to filter out artifacts.

### 2.5. Coimmunoprecipitation

Total protein of BLZ211 cells was used to immunoprecipitate and coimmunoprecipitate interacting proteins (prey proteins) according to the protocol of ProFound Co-Immunoprecipitation (Co-IP) Kit (PIERCE). The purified antibody was put into the spin cup containing the gel for antibody immobilization. The BLZ211 cells were washed once with PBS (Product number 28372; 0.1 M phosphate, 0.15 M NaCl, and pH 7.2). The 200 *μ*L M-PER reagent was added to the plate. Lysate was collected and transferred to a microcentrifuge tube for centrifugation of samples at ~13,000 ×g for 5–10 minutes to pellet the cell debris. The prey complex and controls were put to the appropriate gel in the spin cups, and 0.4 mL of Co-IP Buffer was added. The tubes were inverted 10 times and centrifuged. 100 *μ*L elution buffer was put to the gel in the spin cup and the tube was centrifuged. 5 *μ*L of the 5X sample buffer was added to the sample and applied to the gel for electrophoresis.

### 2.6. LC-MS/MS

The mobile phase buffer A contained H_2_O and 0.1% methanoic acid. The mobile phase buffer B contained acetonitrile and 0.1% methanoic acid, 120 min linear gradient elution, and flowed at 1 *μ*L/min. This consisted of a full mass scan (*m*/*z* 400–2000), zoom scan on the most abundant ion to determine charge state, and a tandem mass spectrometry (MS/MS) scan to collect collision-induced dissociation (CID) spectra on peptides. Automated analysis of CID spectra to determine the amino acid sequence of peptides was performed on computer (SEQUEST software; ThermoFinnigan) as described by Yates III et al. [10].

### 2.7. Immunofluorescence

BLZ211 cells were cultured in 6-well plate with cover slips for 24 hr. The cells on cover slip were fixed for 10 min by immersion in −20°C precooling methanol and washed in phosphate-buffered saline (PBS) three times. Cells were incubated in 0.5% TritonX-100/PBS for 5 min at room temperature and washed in PBS three times and incubated with goat serum for 1 h at room temperature. Cells were incubated for 4 h at 4°C on a rocker platform in 1:10 dilutions of experimental McAb in PBS. The control group uses PBS instead of McAb as primary antibody. Then cells were washed in PBS three times, incubated with secondary antibody for 40 min at 4°C in 1 : 40 dilutions of fluorescein-conjugated goat anti-mouse IgG in a PBS media, followed by incubation with DAPI for 5 min at room temperature, and washed in PBS three times. The cover slips were subjected to another washing cycle before being monitored for specific fluorescence under an immunofluorescence microscope.

### 2.8. Immunohistochemistry Procedures

35 bladder cancer tissues and adjacent normal tissues were used to analyze the expression of Hsp74 with McAb. Paraffin sections of bladder cancer tissues were dewaxed and dehydrated by graded ethanol. Endogenous peroxidase activity was quenched and antigen retrieval was done by 5 min heating in citric acid. After blocking, sections were incubated with Hsp74 McAb at 4°C overnight. Immunoreactivity was detected using horseradish peroxide conjugated second antibody and DAB. Tissue structures were visualized by counterstaining with hematoxylin. Comparisons between groups for bladder cancer tissues and adjacent normal tissues were performed by applying chi-square test as indicated. A *P* value of ≤0.05 was considered significant.

## 3. Results

### 3.1. Monoclonal Antibody

Fusion cells were inoculated in a 96-well plate, in which clone growth appeared in 62 wells. Using immunohistochemistry method, 29% of the 62 clones were found secreting specific antibody. The subcloning with limited dilution assay did not stop until the preliminary screening positive hybridoma cells were 100%.

### 3.2. 2-DE and Western Blot

**Figure 1:**
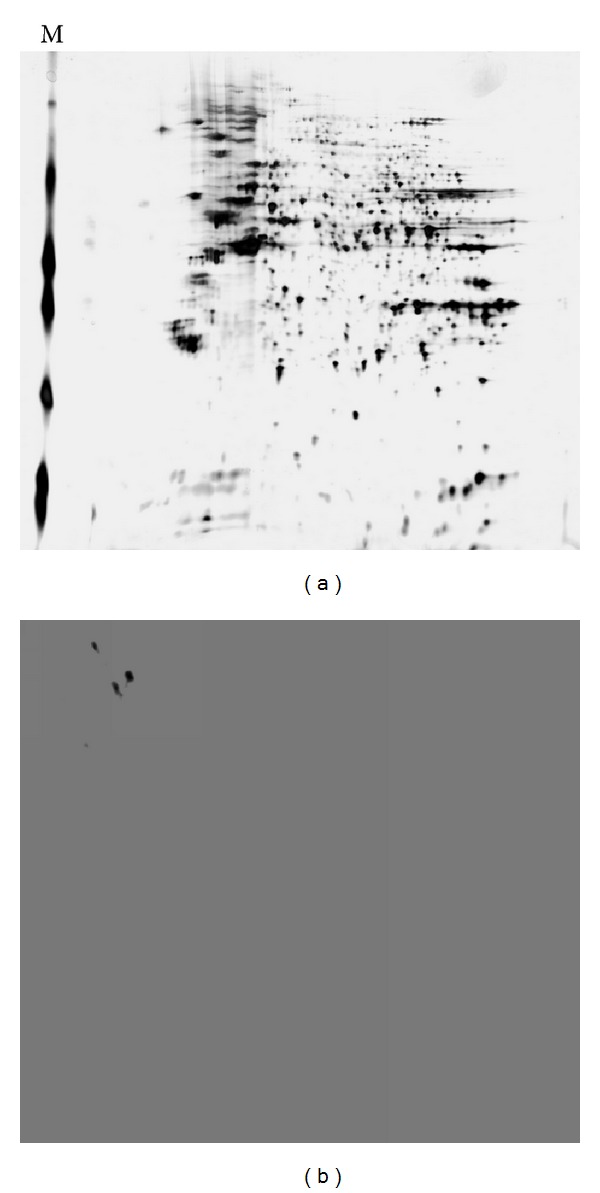
(a) 2-DE gel showing the proteome map of total soluble protein from BLZ211 cells. M: mw markers. (b) Western blot showing 3 spots.

**Figure 2:**
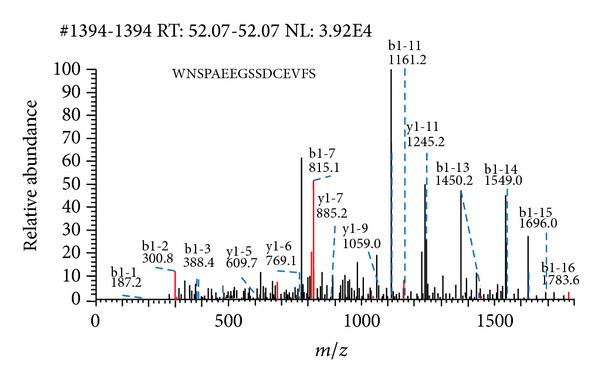
Representative MS/MS spectrum of the peptide of HSP74.

To analyze the proteome of BLZ211 cells, soluble proteins from the BLZ211 cells were separated by 2-DE (Figure 1(a)). Western blot showed 3 protein spots were more intense in BLZ211 cells (Figure 1(b)). One of the protein spots was detected by LC-MS/MS with an apparent mass of 94299.96 Da, score 70.4, accession 6226869, peptides (Hits)7(70000), and pI of 5.18, which was identified as Hsp74 (Figure 2).

### 3.3. Identified Keratin 1

**Figure 3:**
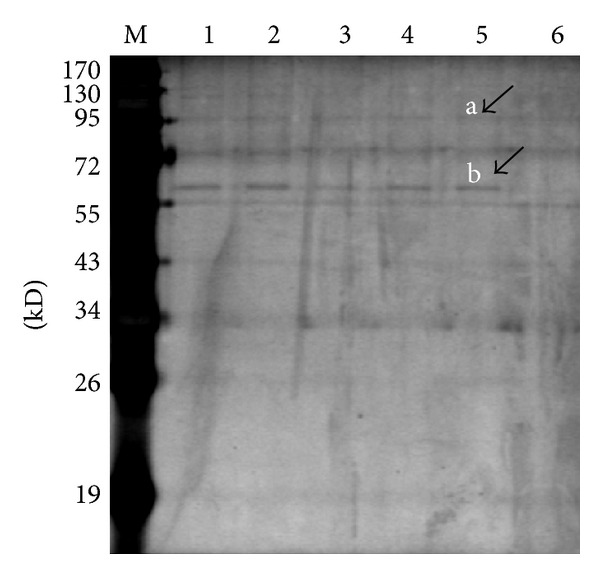
Demonstration of MAb with BLZ211 cells protein reaction by coimmunoprecipitation. M: marker; (a) Hsp74 (b) keratin 1. Lanes 1–5: BLZ211 cells protein; lane 6: negative control; lanes identified by LC-MS/MS.

**Figure 4:**
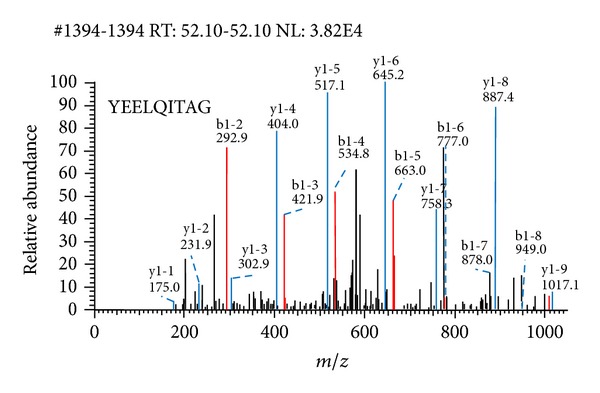
Representative MS/MS spectrum of the peptide of keratin 1.

Keratin 1 (Figure 4) was identified associated with Hsp74 by coimmunoprecipitation (Figure 3), LC-MS/MS. Compared with NCBInr database showed score 198.2, accession 11935049.0, peptides (Hits)20(191000), theory PI 8.16, and MW 66066.74 Da.

### 3.4. Cellular Location

**Figure 5:**
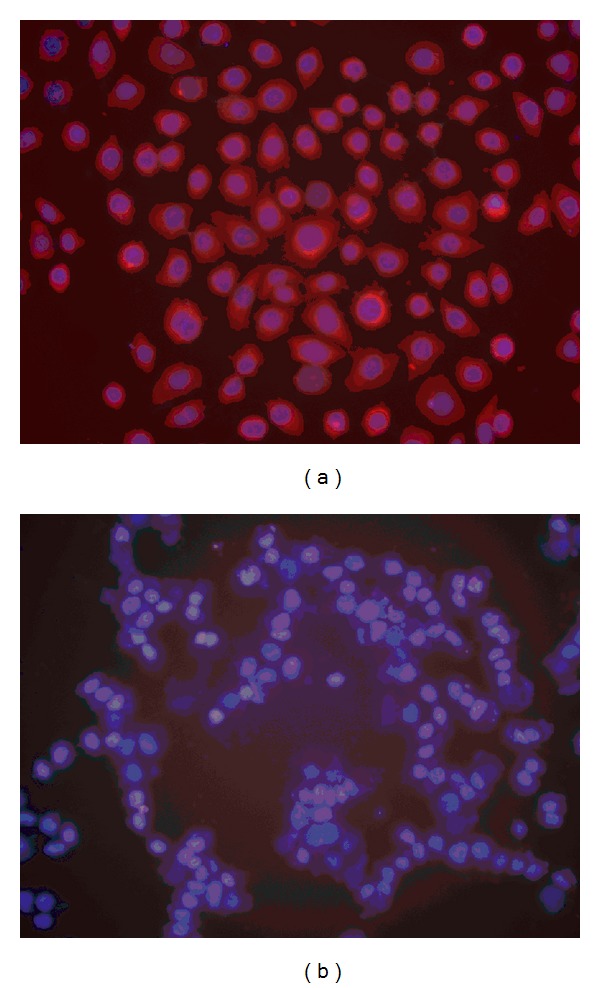
Immunofluorescence of the cellular location analysis of expression products of gene HSP74 showed that they are all distributed in cellular membrane, cytoplasm and nucleus (a), negative control (b) ×200.

The cellular location of expression products of gene Hsp74 was analyzed using immunofluorescence. The results showed that they are all distributed in cellular membrane, cytoplasm and nucleus (Figure 5). The cellular membrane and cytoplasm show all red, and the cellular nucleus shows some red and some blue.

### 3.5. Hsp74 Expression in Tissue

**Figure 6:**
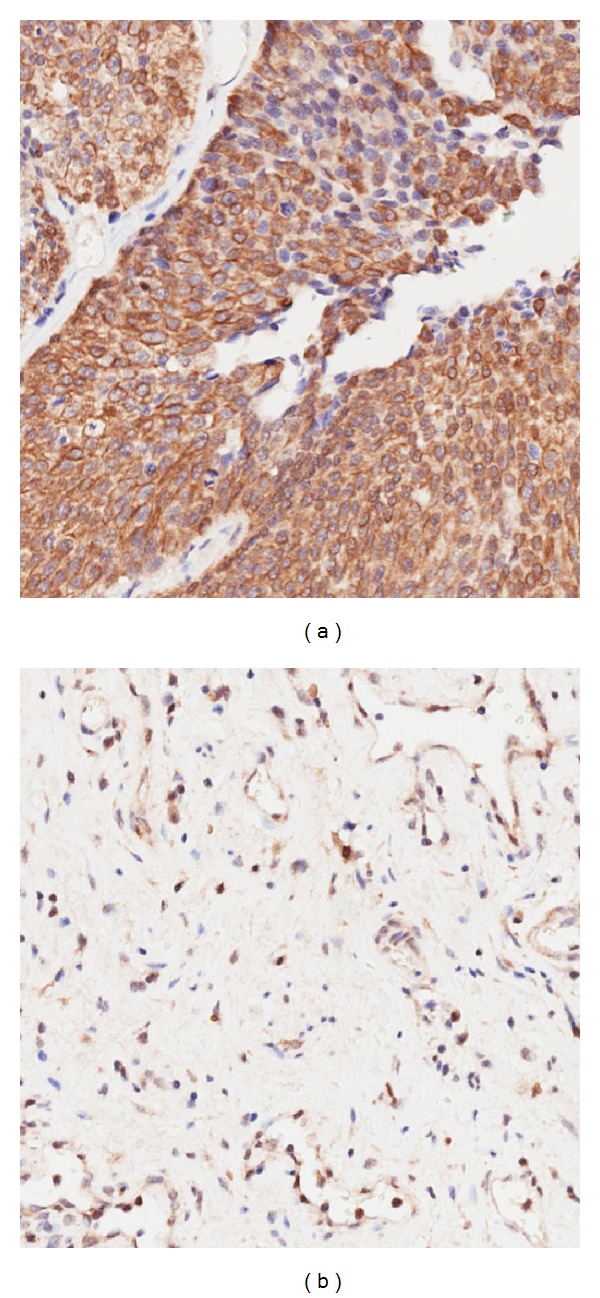
Immunohistochemistry of the tissue location analysis of expression products of gene HSP74 showed that they are distributed positively in bladder cancer tissues (a) and negatively in bladder cancer adjacent normal tissues (b) ×20.

Among the 35 bladder cancer cases, the positive expression rate of Hsp74 in bladder cancer tissues was 74.2%, which was similar to that in adjacent normal tissues (*X* ^2^ = 0.063, 0.75 < *P* < 0.9). But there was a significant difference in the expression intensity distribution between the two groups (*X* ^2^ = 21.86, *P* < 0.005). In most cases, the expression intensity of Hsp74 in bladder cancer tissues was higher than in normal bladder tissue (Table 1, Figure 6).

## 4. Discussion

Bladder cancer is a prevalent disease that causes substantial morbidity and mortality. Despite the continued refinement of surgical techniques, namely, radical cystectomy, the prognosis of muscle-invasive bladder cancer has remained unchanged for the past 30 years with five-year survival rate remaining disappointingly low at 40%. Understanding the biology mechanism underlying tumorigenesis and tumor progression of bladder cancer is essential for improving the capacity to diagnose and treat the disease. Unraveling the biological complexity underlying these processes is expected to provide novel tools of predictive nature and to enable identification of therapeutic targets by selecting those molecular targets significantly and differentially expressed in bladder tumors. Numerous markers that correlate to some extent with bladder cancer stage and prognosis have been identified. However, the ability of most markers in predicting the clinical outcome of individual tumors is limited, and alternative markers are still needed for detection of the disease and for predictive purposes.

We did a related research of bladder cancer using immunoscreening technology previously [11]. Recent advances in expression profiling of cancer cells by proteomic technologies, high-resolution 2-dimensional electrophoresis (2-DE), and mass spectrometry have made it possible to identify candidate proteins as tumor markers in various cancers. In bladder cancer, Celis et al. [12] performed proteome analysis by extensive 2-DE and developed a comprehensive 2-DE database for bladder cancer that includes profiles of both transitional and squamous cell carcinoma. Therefore we used 2-dimensional electrophoresis and mass spectrometry to carry out exploratory research in bladder cancer and identified Hsp74 as potential target. Immunofluorescence analysis showed that Hsp74 protein was distributed in cytoplasm. This result gives us the bladder cancer intracellular localization of Hsp74 in the preliminary concept.

Hsp74 belongs to a member of the family heat-shock (stress) proteins (HSPs). In the face of thermal, chemical, or physiological stresses that cause unfolding of proteins, cells preferentially express HSPs, a process called the heat-shock (or stress) response. Related researches on Hsp can draw the following conclusions: (1) Hsp has relevance with cancer diagnosis; (2) detection of Hsp can help identifying abnormalities in carcinogenesis; (3) Hsp is associated with some tumor differentiation degree; (4) Hsp has significant correlation with some specific molecule. HSP expression is associated with carcinogenesis, cell proliferation, differentiation, and apoptosis. So the detection of Hsp has important significance in the program of individualized cancer therapy, regular followup, and avoidance of excessive use of cytotoxic cancer drug. Research on Hsp provides a new encouraged approach and a new target for immunotherapy [13].

It is common for a constitutively expressed Hsp70 cognate (hsc70) to be present in cells under all conditions, where it functions as a chaperone during normal protein synthesis. As one of Hsp70 isoforms, Hsp74 has been demonstrated to regulate cytoskeletal stability [14]. Mucous membrane of urinary bladder is exposed for longtime to the urine which contains various kinds of mutagenic activity materials and toxic metabolites. Elevated Hsp74 expression might be the result of mucous membrane of urinary bladder chronic stress reaction.

Hsp74 was associated with keratin 1 as determined by coimmunoprecipitation from bladder cancer cell line BLZ211. Keratin 1 belongs to keratin family and is a specific marker of mammal epithelium terminal differentiation since keratin 1 protein mainly locates in epithelial prickle cell layer and epithelial granular cell layer. Monoclonal antibodies to keratin 1 carboxy terminal (synthetic peptide) provide an important means of examining keratin expression in epidermal tumors and keratinizing disorders [15]. Cytokeratins 1, 7, and 14 immunoexpression is helpful in the diagnosis of basaloid squamous carcinoma [16]. It remains to be determined whether the binding of Hsp74 to keratin 1 is dependent on a linear sequence of keratin 1 or a conformation of keratin 1 tetramers or polymers. Furthermore, although our data suggest a direct interaction of Hsp74 with keratin 1, we cannot exclude the possibility that this interaction may be mediated by one or more additional proteins that form a large complex.

To detect tumor mark by monoclonal antibodies is a kind of classical method. Using a combination of techniques including 2-DE, co-IP, western blot, LC-MS/MS, immunofluorescence, and immunohistochemistry, we find the expression intensity of Hsp74 in bladder cancer tissues is higher than that in normal bladder tissue. The results of this research suggest that Hsp74 might be a potential marker of bladder cancer, although further validation is still needed. To use this antigen to detect tumor has more problems which need to be resolved. The relevance of Hsp74 expression with clinical pathological characteristics and survival time is worth to follow-up study. Specifically, in tumor formation the interaction mechanism of keratin 1 and Hsp74 need to be clarified further.

## Figures and Tables

**Table 1:** Hsp74 expression in bladder cancer tissue and adjacent normal tissues.

Pathology	Number	Hsp74 (*n*)				Positive rate (%)
		−	+	++	+++	
Bladder cancer tissue	35	9	4	19	3	74.2
Adjacent normal tissues	35	10	20	5	0	71.4
